# Agreement in the assessment of practical skills in basic CPR between observers and a CPR mannequin with a specific software

**DOI:** 10.1186/2197-425X-3-S1-A749

**Published:** 2015-10-01

**Authors:** B Sánchez, R Algarte, M Cerdà, X Balanzó, JM Giraldo, M Pacheco, R Ferrer, S Quintana

**Affiliations:** Intensive Care Department, Hospital Universitario Mutua de Terrassa, Terrassa, Spain; Consell Català de Resuscitació, Barcelona, Spain

## Introduction

Conducting a training activity usually involves assessing to students the knowledge and/or the skills acquired. Assessment methods must be objectives and representatives of the item being evaluated and able to ensure their independence from the evaluator. The assessment of practical skills in cardiopulmonary resuscitation (CPR) acquired by a student during a training course conforming the quality criteria for CPR on adults (2010 International Guidelines on Resuscitation ^1^), continues to be accomplished currently by observers. Increasingly, other objective methods of assessment (CPR manikin with specific ^2^ software, mechanical devices, etc.) of these skills with an excellent degree of accuracy and feedback, are being extended. The correct evaluation of these practical skills is crucial to ensure their correct acquisition, as a first step to the translation into clinical practice.

## Objectives

To check the agreement between three different observers to each other and with a mechanical device with a specific software for CPR.

## Methods

Descriptive and univariate analysis according to Cohen´s Kappa coefficient and the intraclass correlation coefficient (ICC). 54 volunteers, health workers, with training and experience in CPR, performed a complete sequence of basic CPR maneuvers in a dummy (Laerdal PC v 4.2.1 Skill Reporting Software) (L). Three expert instructors (A, B and C) in teaching courses in CPR evaluated, visually, the performing of correct external chest compressions regarding the placement of the hands, depth compression and decompression, and rate. We analyze the concordance between the observers (A, B and C) among themselves and with L.

## Results

**Figure 1:**
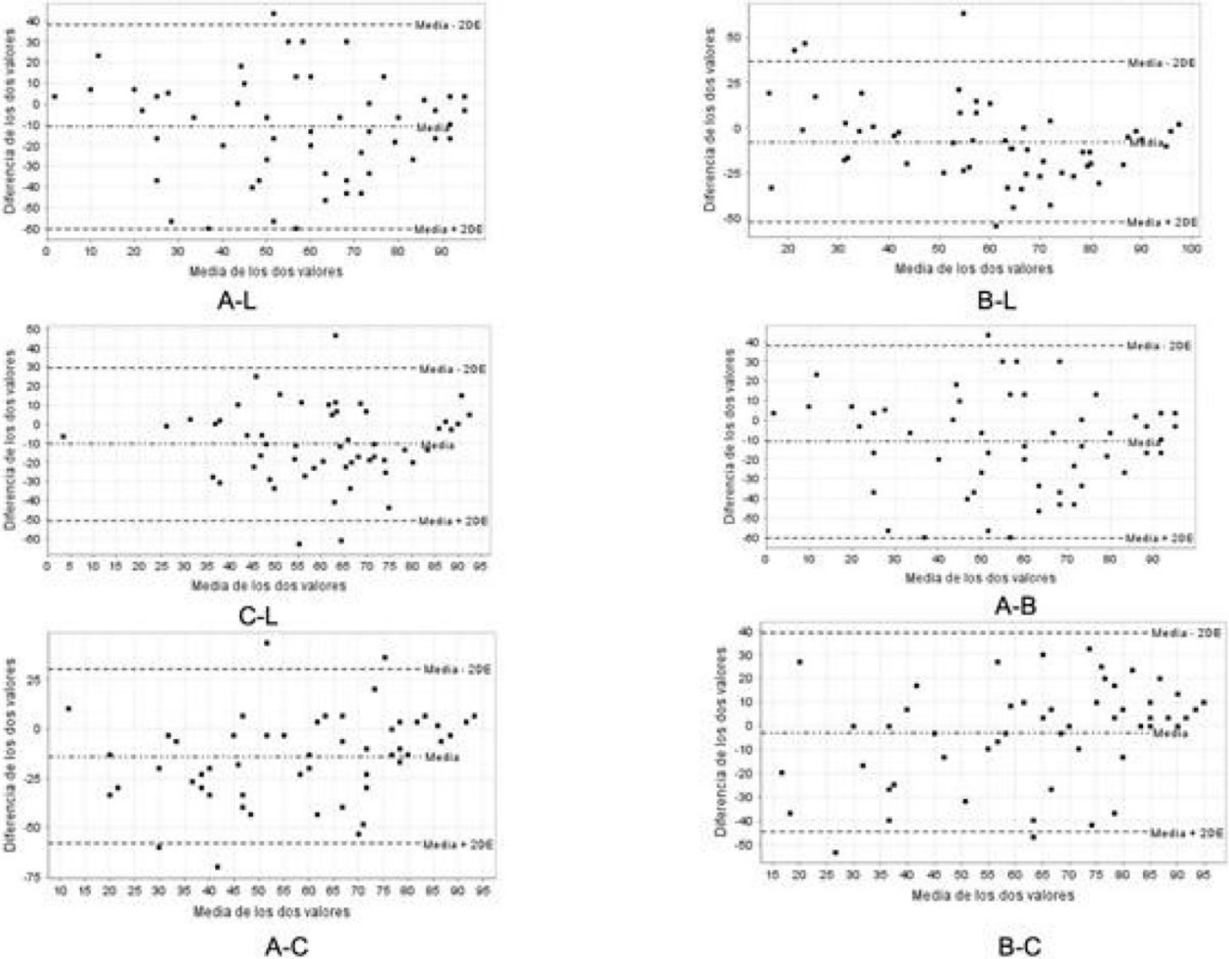
**Bland and Altmann graphics.**

Table 1 shows the mean scores of A, B, C and L for the 54 students. Table 2 shows the crude concordance, the value of Cohen´s kappa coefficient and the ICC. Figure 1 shows the Bland and Altmann graphics. We can observe that the degree of agreement and uniformity among the 4 evaluators are poor, and there is a high degree of dispersion without a defined trend.

**Table 1:** Mean scores

EVALUATORS	MEAN	SD
A	51.8	26.8
B	62.8	27.6
C	65.7	20.3
L	55.1	20.7

**Table 2:** Concordance between pairs of evaluators

EVALUATORS	Crude concordance %	Cohen´s Kappa (K) coefficient	Intraclass correlation coefficient
A-B	78	0.52	0.54
A-C	74	0.45	0.45
B_C	85	0.70	0.43
A-L	74	0.45	0.53
B-L	66	0.35	0.56
C-L	70	0.41	0.44

## Conclusions

The CPR skills assessment in a visual way by expert evaluators is associated with an important lack of agreement and a high dispersion. At present, it must pose routinely the use of mechanical devices for training and evaluation of these skills.
